# Selection of reference genes for quantitative real-time PCR in a rat asphyxial cardiac arrest model

**DOI:** 10.1186/1471-2199-9-53

**Published:** 2008-05-28

**Authors:** Kristina Langnaese, Robin John, Hannes Schweizer, Uwe Ebmeyer, Gerburg Keilhoff

**Affiliations:** 1 Institute of Medical Neurobiology, University of Magdeburg, Leipziger Str. 44, D-39120 Magdeburg, Germany; 2 Department of Anaesthesiology and Critical Care Medicine, University of Magdeburg, Leipziger Str. 44, D-39120 Magdeburg, Germany

## Abstract

**Background:**

Cardiac arrest, and the associated arrest of blood circulation, immediately leads to permanent brain damage because of the exhaustion of oxygen, glucose and energy resources in the brain. Most hippocampal CA1 neurons die during the first week post the insult. Molecular data concerning the recovery after resuscitation are sparse and limited to the early time period. Expression analysis of marker genes via quantitative real-time RT-PCR enables to follow up the remodeling process. However, proper validation of the applied normalization strategy is a crucial prerequisite for reliable conclusions.

Therefore, the present study aimed to determine the expression stability of ten commonly used reference genes (*Actb*, actin, beta; *B2m*, beta-2 microglobulin;*CypA*, cyclophilin A; *Gapdh*, glyceraldehyde-3-phosphate dehydrogenase; *Hprt*, hypoxanthine guanine phosphoribosyl transferase; *Pgk1*, phosphoglycerate kinase 1; *Rpl13a*, ribosomal protein L13A; *Sdha*, succinat dehydrogenase complex, subunit a, flavoprotein (Fp); *Tbp*, TATA box binding protein; *Ywhaz*, tyrosine 3-monooxygenase/tryptophan 5-monooxygenase activation protein, zeta polypeptide) in the rat hippocampus four, seven and twenty-one days after cardiac arrest. Moreover, experimental groups treated with the anti-inflammatory and anti-apoptotic drug minocycline have been included in the study as well.

**Results:**

The microglial marker *Mac-1*, used as a target gene to validate the experimental model, was found to be upregulated about 10- to 20-fold after cardiac arrest.

Expression stability of candidate reference genes was analyzed using geNorm and NormFinder software tools. Several of these genes behave rather stable. *CypA *and *Pgk1 *were identified by geNorm as the two most stable genes 4 and 21 days after asphyxial cardiac arrest, *CypA *and *Gapdh *at 7 days post treatment. *B2m *turned out to be the most variable candidate reference gene, being about 2-fold upregulated in the cardiac arrest treatment groups.

**Conclusion:**

We have validated endogenous control genes for qRT-PCR analysis of gene expression in rat hippocampus after resuscitation from cardiac arrest. For normalization purposes in gene profiling studies a combination of *CypA *and *Pgk1 *should be considered 4 and 21 days post injury, whereas *CypA *and *Gapdh *is the best combination at 7 days. *CypA *is most favorable if restriction to a single reference gene for all time points is required.

## Background

Patho-physiological and biochemical processes during a cardiac arrest, resuscitation, and after restoration of spontaneous circulation are extremely complex, and thus far, poorly understood. Under normothermic conditions brain damage begins to develop after 4–5 min of no-flow [1,2] due to total circulatory arrest, mainly because of the exhaustion of oxygen, glucose and energy resources in brain and other parts of the organism. Eight minutes of asphyxiation – resulting in approximately five minutes of complete none perfusion – causes major to subtotal neuronal damage within the CA1 region of hippocampus, as revealed by haematoxylin-eosin staining [3]. Already eight hours after the insult damaged neurons are characterized by shrunken cell bodies and pyknotic nuclei. Necrotic neurons are partially resorbed within the first week after the insult.

This histologically visible massive remodeling process can be expected to be accompanied with considerable changes in mRNA and protein expression. Only few data are available and these are limited to the early period after the insult. For example the level of the stress related proteins HSP70 and HSP40 is increased 12 h after the ischemic insult [4], as well as the mRNA amount of HSP10, a heat shock protein of the mitochondrial matrix [5]. Caspase-1 and -3 are detectable immunohistochemically 72 h after asphyxia [6]. Moreover, the mRNAs of MMP-9, a matrix metalloproteinase, and TIMP-1, tissue inhibitor 1 of matrix metalloproteinase, are upregulated 6 h after the insult [7]. At 24 h after cardiac arrest BDNF transcripts, namely those containing exons 1 and 3, as well as BDNF protein are increased [8,9].

When attempting to analyze the molecular biological consequences of an ischemic insult due to asphyxial cardiac arrest (ACA), a model of neurological injury after sudden cardiac arrest, real-time quantitative RT-PCR (qRT-PCR) is the method of choice for monitoring alterations of gene expression patterns that accompany the recovery process in the damaged brain. qRT-PCR enables a sensitive and accurate quantification of mRNA expression levels.

However, selection of an appropriate normalization strategy is of crucial importance for data interpretation, because data need to be controlled for the experimental error introduced during the multistage process of isolating and processing RNA [10-12].

The most frequently applied approach for normalization is the use of an internal control or reference gene, often referred to as housekeeping gene. A growing number of recently published articles reflect the need to carefully validate reference genes for each particular experimental model [13-16]. To be used as a suitable reference gene several criteria should be fulfilled. The expression should be stable, not regulated or influenced by the experimental conditions or treatments. In addition, the expression level of the reference gene should be similar to the target genes in the analyzed samples. The amplification of the reference gene should be RNA-specific. The importance of choosing a reliable reference gene is underlined by the fact, that the use of an unstable reference gene for normalization will obscure real changes or produce artificial changes in gene expression [13,17]. Therefore, the validation of reference genes for each experimental situation is a crucial requirement for the acquisition of biological meaningful data [10,18,19].

The aim of this study was to identify the most stable reference gene, or a combination of the most stable genes, in rat hippocampus during the long term recovery after global hypoxic ischemia. We therefore evaluated ten commonly used housekeeping genes for their change in the ACA experimental model at 4, 7 and 21 days after the insult. The analysis was conducted in parallel also with experimental groups that received treatment with minocycline. Minocycline is a semisynthetic second-generation tetracycline with broad-spectrum antimicrobial activity, which has been used for decades in humans to treat infectious diseases [20]. In addition to its antimicrobial actions minocycline was shown to exert beneficial effects in a variety of models of neurological disorders (reviewed in [21-24]). This application area relies on minocycline's anti-inflammatory and anti-apoptotic properties. For example minocycline has been demonstrated to reduce infarct size after focal cerebral ischemia [25-28], to inhibit ischemia induced activation of microglia and to prevent upregulation of ischemia-induced mRNAs [25,29]. Therefore, the analysis of minocycline's effectiveness is desirable in the ACA experimental model too. However, since such a pharmacological treatment might up- or downregulate not only target genes, but also housekeeping genes, the respective experimental groups were already included in this study.

Our results will provide information about appropriate reference genes for the normalization of qPCR data during long time recovery studies in the cardiac arrest model necessary for future gene expression studies.

## Results

Ten candidate reference genes were selected from commonly used control genes. Genes with different functions were chosen to avoid genes belonging to the same biological pathways that might be coregulated (see Tab. 1 and 2 for gene names and function).

**Table 1:** Primer sequences and amplicon characteristics

Gene	Sequence	Reference ^a^	Position cDNA ^b^	Position gene	Amplicon length (bp)	Product TM^e, f ^(°C)	PCR efficiency^f ^(%)
Gapdh	5'-CAACTCCCTCAAGATTGTCAGCAA-3'	NM_017008	493–516	Int. span.	118	82.9	105
	5-'GGCATGGACTGTGGTCATGA-3'	NC_005103	610-591				
*Actb *^c^	5'-AAGTCCCTCACCCTCCCAAAAG-3'	V01217	3474–3495	Exon	97	82.9	92.5
	5'-AAGCAATGCTGTCACCTTCCC-3'	V01217	3570-3550				
*CypA *^c^	5'-TATCTGCACTGCCAAGACTGAGTG-3'	M19533	381–404	Int. span.	126	82.0	98
	5'-CTTCTTGCTGGTCTTGCCATTCC-3'	NW_047430	507-485				
*B2m*	5'-CGAGACCGATGTATATGCTTGC-3'	NM_012512	286–307	Int. span.	114	79.8	92
	5'-GTCCAGATGATTCAGAGCTCCA-3'	NC_005102	399-378				
*Rpl13a*	5'-GGATCCCTCCACCCTATGACA-3'	NM_173340	334–354	Int. span.	132	83.5	100
	5'-CTGGTACTTCCACCCGACCTC-3'	NC_005100	464-444				
*Hprt *^c^	5'-CTCATGGACTGATTATGGACAGGAC-3'	NM_012583	179–203	Int. span.	123	80.9	97
	5'-GCAGGTCAGCAAAGAACTTATAGCC-3'	NW_048050	301-277				
*Ywhaz*	5'-GATGAAGCCATTGCTGAACTTG-3'	NM_013011	955–976	Int. span.	117	77.6	95
	5'-GTCTCCTTGGGTATCCGATGTC-3'	NC_005106	1071-1050				
*Sdha*	5'-TCCTTCCCACTGTGCATTACAA-3'	NM_130428	1222–1243	Int. span.	105	81.4	98
	5'-CGTACAGACCAGGCACAATCTG-3'	NC_005100	1326-1305				
*Pgk1*	5'-ATGCAAAGACTGGCCAAGCTAC-3'	NM_053291	969–990	Int. span.	104	81.9	99
	5'-AGCCACAGCCTCAGCATATTTC-3'	NC_005120	1072-1051				
*Tbp*	5'-TGGGATTGTACCACAGCTCCA-3'	NM_001004198	679–699	Int. span.	131	78.2	100
	5'-CTCATGATGACTGCAGCAAACC-3'	NC_005100	810-789				
*Mac-1*^d^	5'-CTGCCTCAGGGATCCGTAAAG-3'	NM_012711	680–700	Int. span.	150	79.8	101
	5'-CCTCTGCCTCAGGAATGACATC-3'	NW_047562	829-808				

^a ^Genebank accession number of cDNA (upper line) and genomic sequences or contigs (lower line), ^b ^Position of amplification product within cDNA sequence (genomic sequence for *Actb*), Exon; both primers bind to the same exon, Int. span.; primers bind on different exons, ^c ^Primer sequences according to [46], ^d ^Primer sequences according to [47], ^e ^melting temperature of specific PCR product, ^f ^calculated by MxPro Mx3005P v3.00 software (Stratagene, La Jolla, CA)

**Table 2:** Name and function of the genes

Symbol*	Gene name*	Function
Gapdh	glyceraldehyde-3-phosphate dehydrogenase	Glycolytic enzyme
Actb	actin, beta	Cytoskeletal structural protein
*CypA *(synonym *Ppia*)	cyclophilin A (peptidyl prolyl isomerase A)	Catalyzes the cis-trans isomerization of proline imidic peptide bonds in oligopeptides, accelerating folding
*B2m*	beta-2 microglobulin	Beta-chain of major histocompatibility complex class I molecules
*Rpl13a*	ribosomal protein L13A	Structural component of the large 60S ribosomal subunit
*Hprt*	hypoxanthine guanine phosphoribosyl transferase	Purine synthesis in salvage pathway
*Ywhaz*	tyrosine 3-monooxygenase/tryptophan 5 -monooxygenase activation protein, zeta polypeptide	Signal transduction by binding to phosphorylated serine residues on a variety of signalling molecules
*Sdha*	Succinat dehydrogenase complex, subunit a, flavoprotein (Fp)	Electron transporter in the TCA cycle and respiratory chain
*Pgk1*	phosphoglycerate kinase 1	Glycolytic enzyme
*Tbp*	TATA box binding protein	General RNA polymerase II transcription factor

*according to Entrez Gene database [54]

Using real-time PCR we evaluated the expression of these candidate reference genes in rat hippocampus under four different experimental conditions: sham operated animals, sham operated animals with minocycline treatment, ACA and ACA with minocycline treatment. Each of the four experimental groups was analyzed 4 days, 7 days and 21 days after the insult.

### Quality assessment of qPCR protocol and qPCR efficiency

Agarose gel electrophoresis of PCR products during the initial optimization experiments revealed single bands for all primer sets (not shown). Moreover, melting curve analysis was performed after each run. This always demonstrated a single homogenous melt peak, confirming specific amplification. The melting temperatures of all PCR products are given in Tab. 1. Since PCR products of identical size and melting temperature may also arise due to the existence of processed pseudogenes on the genomic DNA [30], which potentially contaminates the samples, additional control experiments were performed. Indeed, several primer pairs (*Gapdh*, *Actb*, *CypA*, *Pgk1 *and *Rpl13a*) resulted in amplification products of equal size and melting temperature with genomic DNA as template, whereas the other primer pairs did not yield any signal under these conditions. Therefore, though all primer pairs (except for *Actb*) span introns, minus RT controls were performed for the respective primer pairs. No signals were detected in the minus RT controls. Calibration curves were generated using relative concentrations vs. the threshold cycle (Ct). The RSq value (R^2^, linear correlation coefficient), an indicator of fit for the standard curve plotted to the standard data points of all genes ranged from 0.995 to 1.000. Based on the slopes of the standard curves, the amplification efficiencies ranged from 92 % to 105 % (Tab. 1), (derived from the formula PCR efficiency = 10 ^1/slope ^-1, calculated by the Mx3005P software). Efficiencies higher than 100 % may result from this calculation method, which is an overestimate of the "real efficiency" [31].

### Determination of the stability of housekeeping genes by GeNorm and NormFinder

**Figure 1:**
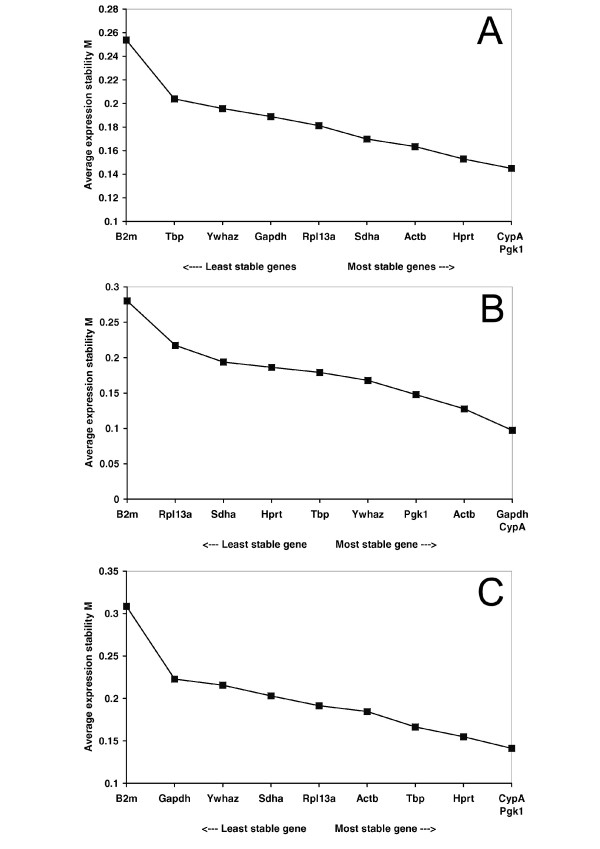
**Gene expression stability of candidate reference genes**. The geNORM program was used to calculate the average expression stability values (M) across all four different treatment groups together at 4 days (A), 7 days (B) and 21 days (C) after ACA treatment. *B2m *is the least stable gene (highest M value) at all three time points. The combination of *CypA*/*Pgk1*, *CypA*/*Gapdh *and *CypA*/*Pgk1 *are the most stable genes at 4 days, 7 days and 21 days, respectively.

**Figure 2:**
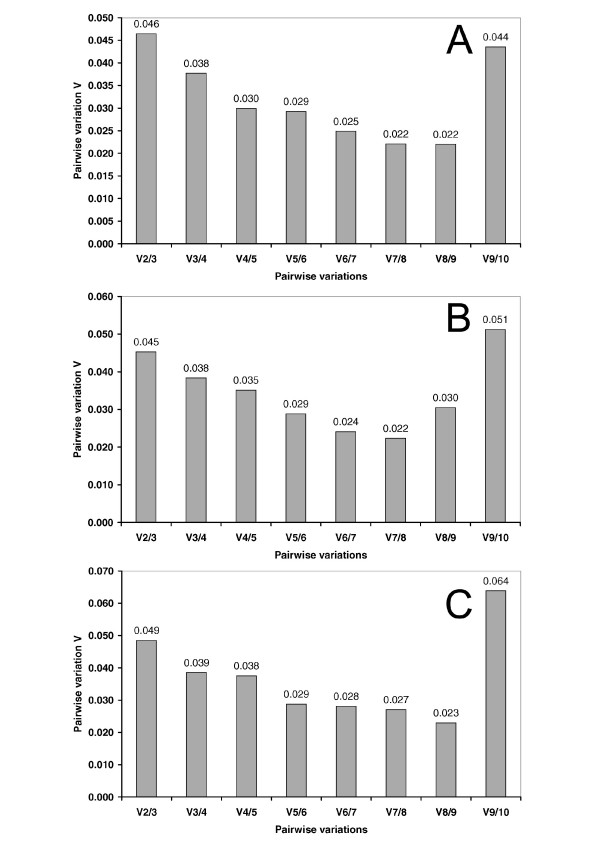
**Determination of the optimal number of control genes**. The geNORM program was used to analyze the pairwise variation between the normalization factor NF_n _and NF_n+1_. Every bar represents change in normalization accuracy when stepwise adding more endogenous controls according to ranking in Fig. 1. Data were analyzed for the three different time points 4 days (A), 7 days (B) and 21 days (C) after ACA. The use of the two most stable genes is in each case sufficient for an accurate normalization (cutoff 0.15 according to [19]). The higher V9/10 is due to the inclusion of a relative unstable gene, *B2m*, and is in accordance with the average expression stability M.

GeNorm was used to identify the most stable reference gene for each of the three analyzed time points separately. Fig. 1 shows the average expression stability values M of the remaining control genes. GeNorm identified *CypA *and *Pgk1 *as the most stable pairwise combination of reference genes for the experimental groups four days after ACA treatment (M value for combination of best two genes 0.145), *CypA *and *Gapdh *for the groups 7 days after the insult (M value for combination of best two genes 0.097) and *CypA *and *Pgk1 *for the 21 day time point (M value for combination of best two genes 0.141). Interestingly, for all the analyzed time points *B2m *shows the highest M value (generated using all ten genes 0.454, 4 days; 0.531, 7 days; 0.651, 21 days). Moreover, we analyzed the pairwise variation values (V) between two sequential normalization factors containing an increasing number of genes (Fig. 2). The pairwise variation value of V2/3 is only 0.046 for the experimental group 4 days after ACA, 0.045 for the 7 days experimental groups and 0.049 for the 21 days groups. According to Vandesompele et al. [19] the ideal pairwise variation value is less than 0.15. Thus, although including further reference genes the V value further decreases, there is no need to include more than two genes into the normalization factor, because this would not improve normalization dramatically.

When NormFinder was used to analyze the same data set a slightly different order of gene stability was calculated (Tab. 3). More stable gene expression is indicated by lower average expression stability values. For the experimental groups 4 days after ACA treatment *Actb *was identified as the most stable gene with a stability value of 0.035. For the groups 7 days after ACA *Gapdh *was calculated to be the most stable gene with a stability value of 0.032 and for 21 days *Tbp *with 0.027. Interestingly, *B2m *was identified to be the most unstable gene with stability values of 0.132, 0.117 and 0.126 at 4 days, 7 days and 21 days after ACA, respectively.

**Table 3:** Stability values of HKGs revealed by NormFinder

Gene name	4 d	7 d	21 d
*Actb*	**0.035**	0.039	0.033
*B2m*	0.132	0.117	0.126
*CypA*	0.053	0.050	0.039
*Gapdh*	0.062	**0.032**	0.064
*Hprt*	0.070	0.061	0.066
*Pgk1*	0.057	0.055	0.048
*Rpl13a*	0.049	0.062	0.039
*Sdha*	0.042	0.037	0.048
*Tbp*	0.060	0.036	**0.027**
*Ywhaz*	0.065	0.045	0.068
			
Best gene	Actb	*Gapdh*	*Tbp*

**Figure 3:**
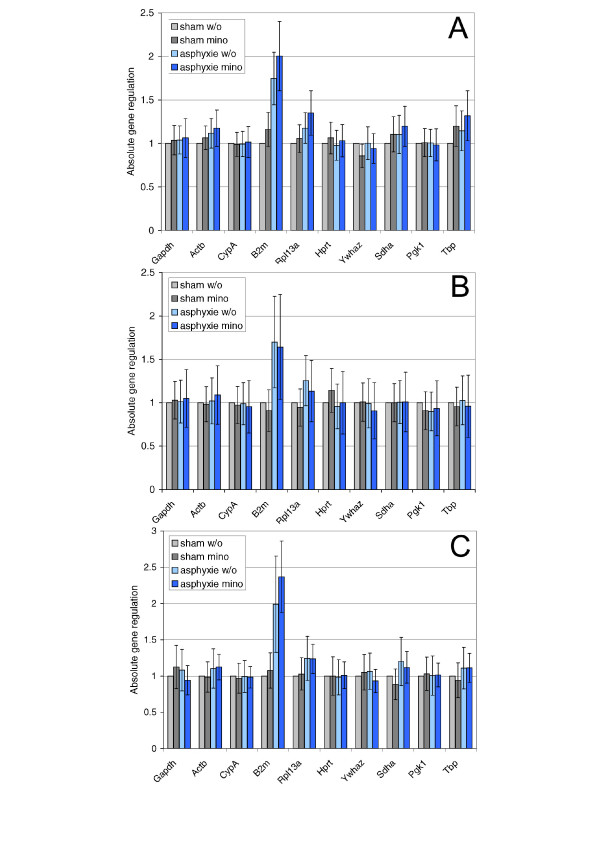
**Expression analysis of ten potential reference genes in the ACA model**. Expression ratios were calculated using REST-MCS. The absolute gene regulation values along with the corresponding standard error were used to create the graph. The control group, i.e. sham operated, was assigned a value of 1.

After having identified the most stable combination of two reference genes by geNorm, we calculated the expression of the other genes for each of the three different time points. Data were normalized to the normalization factor calculated by geNorm, taking into account the combination of the two best performing housekeeping genes. Most of the analyzed candidate reference genes are not significantly changed by either of the treatments at any of the three time points (Fig. 3).

**Figure 4:**
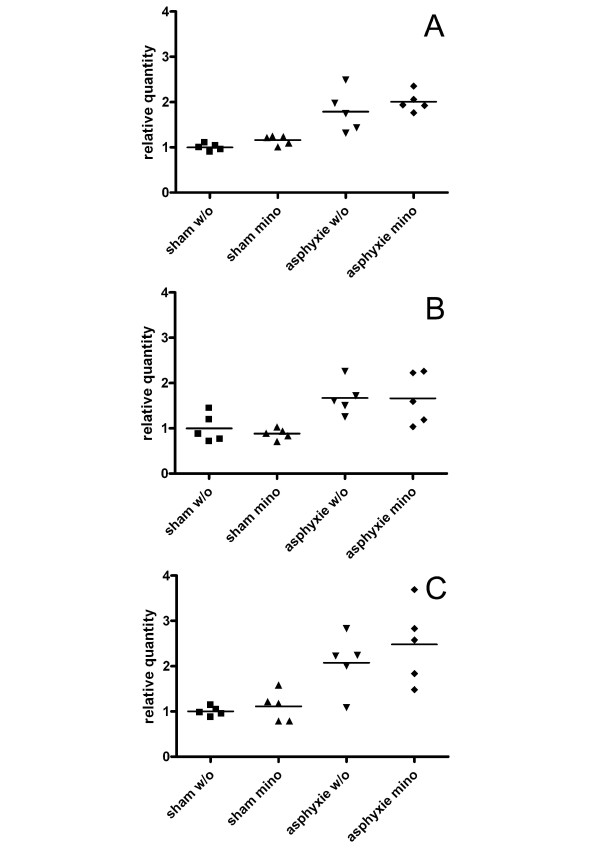
***B2m *is unsuitable as an endogenous control gene after ACA treatment**. Scatterplots showing *B2m *mRNA levels in the different treatment groups.*B2m *expression in hippocampus is upregulated due to asphyxial cardiac arrest 4 days (A) and 21 days (C) after treatment. Though not significant, there is a tendency for *B2m *upregulation 7 days after ACA too (B). Compared to the sham treated group *B2m *expression is 1.7 ± 0.30 -fold upregulated in the ACA group (p-value 0.038) and 2.0 ± 0.39 -fold upregulated in the ACA with minocycline treatment group (p-value 0.009), respectively four days after the injury. Twenty-one days after treatment *B2m *is 2.4 ± 0.49 -fold upregulated in the ACA with minocycline treatment group (p-value 0.020) vs. sham operated group. Absolute gene regulation value, standard error and p-value are given as calculated using REST-MCS software.

In contrast, *B2m *transcript, which was identified as the most unstable gene by geNorm as well as by NormFinder, is 1.7-fold upregulated in the ACA group and twofold upregulated in the ACA with minocycline treatment group 4 days after treatment compared to the sham treated group. At 21 days after treatment there is a 2.4 fold upregulation in *B2m *expression in the ACA with minocycline treatment group vs. control. Though not significant, there is a trend for *B2m *mRNA upregulation at 7 days after treatment too (Fig. 4).

### Assessment of a microglial activation marker

**Figure 5:**
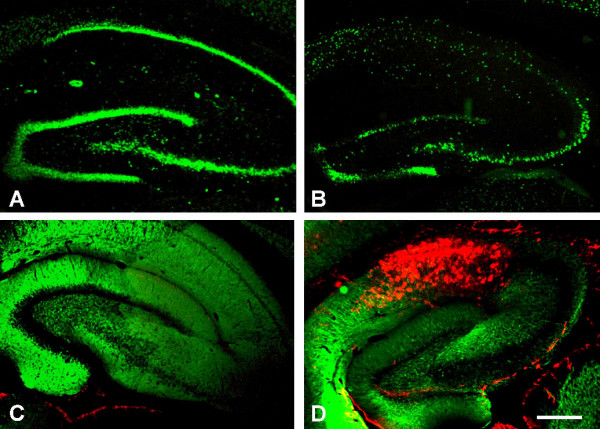
**Immunofluorescence staining of the hippocampus**. In sham-operated animals (A), the hippocampus appeared intact on NeuN immunostaining 21 day post insult. Asphyxia-exposed animals (B), however, showed a significant decrease of NeuN immunopositive cells, particularly prominent in the CA1 and dentate gyrus regions. In parallel, OX42-immunocytostaining (red) revealed a massive microgliosis in the CA1 region (D) when compared to sham-operated animals (C). C, D: MAP2 counter stained, bar = 400 μm.

Using immunohistochemistry, NeuN staining is significantly reduced due to ACA compared to sham-operated animals (Fig. 5A, B). This reflects the neuronal damage within the CA1 and dentate gyrus regions. The neuronal loss is paralleled by massive microgliosis, as demonstrated by OX42 immunostaining (Fig. 5C, D).

**Figure 6:**
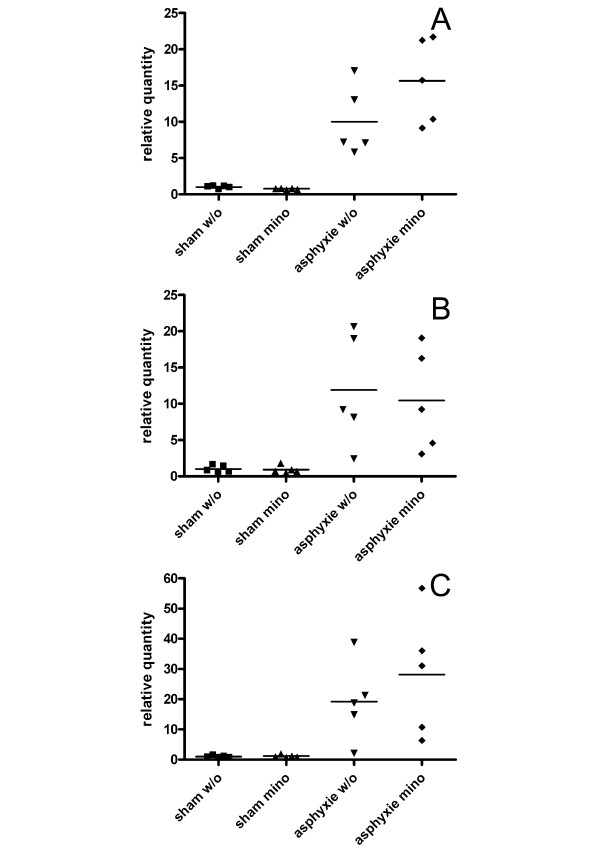
***Mac-1 *is upregulated after ACA treatment**. Scatterplots showing *Mac-1 *mRNA levels in the different treatment groups. Compared to the sham treated group the expression of the microglia marker *Mac-1 *is 9.3 ± 2.34 -fold upregulated four days after the insult in the asphyxial cardiac arrest group (p-value 0.006) and 14.9 ± 4.56 -fold in the ACA with minocycline treatment group (p-value 0.001) (A). Seven days after treatment *Mac-1 *is 10.3 ± 6.18 -fold (p-value 0.011) and 9.2 ± 4.84 -fold (p-value 0.001) upregulated in the ACA and in the ACA with minocycline treatment groups, respectively (B). The increased *Mac-1 *expression is detectable 21 days after the insult too (14.3 ± 9.17-fold, p-value 0.024 and 22.2 ± 9.93-fold, p-value 0.006) in the ACA and ACA with minocycline treatment groups, respectively (C). Calculations were done using REST-MCS software.

Moreover, in order to evaluate our experimental paradigm also via qRT-PCR, we analyzed the expression of the microglial marker *Mac-1 *(synonymous names *Cd11b *or *Itgam*). This gene is constitutively expressed by resting microglia as well as by macrophages and is known to be upregulated upon microglia activation [32]. We found an about 10- to 20-fold upregulation of *Mac-1 *four days after the ACA insult which persisted during the later time points analyzed (Fig. 6).

## Discussion

The reliability of qRT-PCR data will be greatly improved by inclusion of a reference gene which passed all steps of the analysis similarly to the gene to be quantified [10]. This normalization corrects for variations caused e.g. by errors in sample quantification, RT efficiency differences or cDNA sample loading variation.

The transcription level of a good endogenous reference gene should be invariable by the different experimental conditions since the use of an unstable gene may cause erroneous results, either obscuring real changes or produce artificial changes of the target genes [17,33].

The present study is the first detailed survey on the stability of rat housekeeping genes after a hypoxic ischemic insult due to ACA. Ten commonly used candidate reference genes were analyzed. Our observation of pseudogene-driven amplification from genomic DNA during the initial primer evaluation highlights a general need to control for these possible artifacts by including Minus RT-control reactions, even when intron-spanning primers are used [34].

Using NeuN immunocytochemistry, we clearly demonstrated the neuronal loss due to ACA, which is in line with earlier data, based on haematoxylin-eosin staining [3]. This is paralleled by massive microgliosis, as demonstrated by OX42 immunocytochemistry (Fig. 5). Moreover, we proved by qRT-PCR that major gene expression changes indeed occur due to the experimental treatment, since *Mac-1 *was found to be upregulated 10 to 20-fold in the asphyxia groups compared to the control group (Fig. 6). *Mac-1 *is expressed by microglia cells, where it is known to be upregulated due to brain damage. In addition, *Mac-1 *is expressed by macrophages invading into the brain [32].

Concerning the candidate reference genes, our results demonstrate that variations in expression of these genes do exist. Using geNorm, *CypA *and *Pgk1 *were identified as the two most stable genes after four and 21 days, *CypA *and *Gapdh *after seven days post treatment (Fig. 1). Because this software relies on pairwise comparisons it results in a combination of the, at least, two most stable genes. The average expression stability values M generated by stepwise exclusion of the most variable gene do not show a steep, but moderate decrease. This indicates that the variability of most reference gene expression levels between the samples in the experimental groups is rather small. Paralleling this, the calculated pairwise variation values (V) for the inclusion of a third reference gene (V2/3) are 0.046, 0.045 and 0.049 at four, seven and 21 days after cardiac arrest, respectively (Fig. 2). This is well below 0.15, the threshold set by Vandesompele et al. [19], below which further adding of reference genes would not improve normalization. Thus, normalization with a combination of the two best performing genes is sufficient in our experimental setup.

Importantly, *B2m *is clearly identified to be the most unstable gene for all three time points analyzed.

The alternative program, NormFinder, ranks housekeeping genes according to their expression stability using a model-based approach [11]. The program estimates both, the intra- and the inter-group expression variation and calculates candidate gene stability values. The resulting order of genes is not identical to the geNorm output, a fact that is not unexpected since both programs rely on different mathematical approaches [35]. Since the stability values of the genes ranking on the subsequent positions behind the most stable gene do not differ to a great extent, these genes are nearly as stable as the best gene. Interestingly, *B2m *is identified to be the most unstable gene with NormFinder (Tab. 3). Thus, both tools clearly identify *B2m *as the most variable gene in our experimental setup, which should not be used for normalization purposes in this experimental paradigm.

These results are somewhat unexpected, since hypoxic ischemic insults lead to neuronal loss, glial proliferation and the influx of leukocytes in the relevant brain regions [36,37]. Therefore we initially suspected that these major disturbances of the cell populations lead not only to expression changes in multiple target genes, but also to an up- or down regulation of genes that are assumed to be relatively stable in expression under normal circumstances. These genes are often referred to as housekeeping genes. Several studies, performed predominantly in different focal ischemia models, support this assumption. It has been shown, that the expression for example of *Actb*, *Gapdh *or *CypA*, commonly used control genes, varies considerably, depending on the model used and the time points analyzed. For example, a middle cerebral artery occlusion model of focal ischemia alters *Actb *transcript level [38,39], *Gapdh *mRNA amount [40] or both [41,42]. Changes in *Gapdh *and *Actb *expression were demonstrated also in a mouse model of transient forebrain ischemia [43] as well as in a rat model of global cerebral ischemia [44] using microarrays. Contradictory to the above findings, reporting variations in housekeeping gene expression after various ischemic insults, Meldgaard et al. [45] demonstrated *Gapdh *as well as *Hprt *to be reasonable stable in four neurological disease models. Altogether, this demonstrates that a careful screen for reliable reference genes is indispensable for each individual experimental situation.

It is interesting to note, that the spread of data points originating from the individual animals is much wider for *Mac-1 *as well as *B2m *in the asphyxia groups, than for the other analyzed genes in all experimental groups (Figs. 4, 6). *Mac-1*, the microglia marker is clearly upregulated, mirroring activation/proliferation of these cells in the injured brain after ACA. However, as seen by the spread of data points, the extent of upregulation differs markedly between the individuals. One may speculate that each animal performs slightly different to the ACA procedure, for example due to the individuality in the vascular network of the brain. This may lead to the gradually different extent of gene expression changes of regulated genes, whereas unregulated genes do not show this variation.

## Conclusion

Taken together, our data provide a guideline helping to choose reference genes for the analysis of long term gene expression changes due to ACA. Our results suggest the use of the geometric mean of *CypA *and *Pgk1 *four and 21 days after treatment and *CypA *and *Gapdh *at seven days, respectively. These combinations seem appropriate for the detection of slight changes. On the other hand, due to higher costs and efforts using more than one reference gene in multiple samples, only a single reference gene may be preferred. This decision depends on the degree of accuracy required. Based on our data, *CypA *could best be used as an internal reference gene under such circumstances. Furthermore, our data confirm that suitable reference genes are highly specific for a particular experimental situation, thus requiring a careful evaluation for every individual experimental setup.

## Methods

### Asphyxial cardiac arrest model

All animals were maintained in accordance with the guidelines of the German Animal Welfare Act. The study was approved by the Animal Care and Use Committees of the State of Saxony-Anhalt and the University of Magdeburg under the permit number G/1/06. The animals were housed under temperature -controlled conditions at 21 ± 1°C, a 12 h light/dark cycle, and free access to standard rat chow (Altromin 1324™, Altromin GmbH, Lage, Germany) and water.

Sixty age-matched (15 weeks, 300–350 g) and strain-matched (Wistar, inbred, Harlan-Winkelmann; Borchen, Germany) male rats were divided into four treatment groups: (i) asphyxial cardiac arrest (ACA); (ii) ACA with minocycline treatment; (iii) sham operated; (iv) sham operated, with minocycline treatment. Three different survival times were analyzed per group: 4 days, 7 days and 21 days. Thus, 5 animals were used per treatment and time point.

The surgery protocol has been described in detail previously by Ebmeyer et al. [3] with the slight modification that resuscitation was started exactly after 5 min of asphyxiation. During preparation, the insult and the first hour post return of spontaneous circulation (ROSC) body temperature was controlled and maintained at 37°C. Rats were then further kept at normal body temperature (37°C) by placing them in an incubator cage for 24 h post-resuscitation. Blood pressure values were measured according to the protocol. The mean artery pressure (MAP) values were similar in all animals included in the study and the course over time closely resembled the published data for intra-insult temperature-controlled Wistar rats [3]. Animals that showed critical low blood pressure levels (MAP < 50 mmHg) were excluded from further analysis.

Minocycline hydrochloride (Sigma, St. Louis, USA) was administered twice a day, i.p., at a dosage of 6 mg every 12 hours for a period of 5 days. First minocycline injection was performed 60 min after ROSC. The drug was dissolved in PBS at a concentration of 6 mg/ml. Between the injections the solution was stored at 6°C for five days. The volume to be injected was adapted to room temperature before the injection. Sham operated animals received a PBS injection (1 ml).

Rats were sacrificed by decapitation during deep anesthesia. Brains were quickly removed and, for qRT-PCR, hippocampus was dissected on ice, immediately frozen on dry ice and stored at -80°C until RNA extraction.

### Immunohistochemistry

For immunohistochemistry, brains were quickly removed from the cranium, postfixed in 4 % buffered paraformaldehyde (pH 7.4) at 4°C overnight, cryoprotected in a solution of 30 % sucrose (Merck) in 0.4 % buffered paraformaldehyde (pH 7.4) for 2 days and rapidly frozen at -20°C using 2-methylbutan (Roth, Karlsruhe, Germany). Serial sagittal sections (20 μm thick) were cut on a cryostat (Jung Frigocut 2800 E, Leica, Bensheim, Germany). Free-floating sections were washed and incubated with monoclonal mouse anti-NeuN (Chemicon, Temecula, CA, 1:100), polyclonal rabbit anti-MAP 2 (microtubule-associated protein 2, Chemicon 1:2.500), or monoclonal mouse anti-CD 11b/c (OX42, PharMingen, Hamburg, Germany, 1:800) in PBS with 0.3 % Triton X-100 and 1 % normal goat serum overnight at 4°C. Following incubation with primary antibodies, slices were washed in PBS (3 × 5 min), and incubated overnight with the respective secondary antibodies (1:500, goat anti-mouse-IgG Alexa Fluor 488, goat anti-rabbit-IgG Alexa Fluor 488, goat anti-mouse-IgG Alexa Fluor 546, Molecular Probes, Göttingen, Germany), mounted and examined on a fluorescence microscope (Axiophot, Zeiss). Control reactions (substitution of the primary antisera by phosphate buffered saline) yielded negative results (i.e. no specific immunostaining was seen in these sections).

### Reference gene selection and primer design

Candidate reference genes were selected from those most commonly used in the literature. To reduce the chance that these genes might be coregulated, ten genes belonging to different functional classes were selected. Primers for *Actb*, *CypA *and *Hprt *were as published by Peinnequin et al. [46]. *Mac-1 *primers were as published by Raghavendra et al. [47]. All other primers were designed by the primer 3 software [48] based on rat sequences in the database [49]. Here primers were chosen, that bind to different exons in order to avoid non-specific product formation from potentially contaminating genomic DNA. The specificity of the primers was checked using a BLAST search against nucleotide collection (nr) of the NCBI database.

All primers were synthesized by MWG Biotech (Ebersberg, Germany). The sequences of primers are listed in Tab. 1.

### RNA isolation and reverse transcription

Hippocampal tissue was homogenized in peqGOLD TriFast (Total RNA Isolation Reagent, PeqLab, Erlangen, Germany) using an Ultra-Turrax Homogenizer (IKA Labortechnik, Staufen i. Br., Germany). Total RNA was prepared according to the manufacturers instructions. The concentration of total RNA was determined by measuring the optical density at 260 nm and the purity was checked as the 260 nm/280 nm ratio with expected values between 1.8 and 2.0. The integrity of total RNA was assessed by electrophoresis on 1.2 % (w/v) agarose gels. To remove potential DNA contamination, the RNA samples were DNase treated (TURBO DNA-free Kit, Ambion, Austin, USA). First strand cDNA was prepared from 1 μg DNase treated total RNA in a total volume of 20 μl using the RevertAid First Strand cDNA Synthesis Kit (Fermentas, St. Leon-Roth, Germany). Oligo(dT)_18 _primers were used and all samples were stored at -80°C until further analysis. Additionally, cDNA was prepared from a mixed RNA sample (later referred to as pool cDNA) for each of the tree time points. For this purpose equal amounts of DNase treated total RNA of all twenty samples of each time point were mixed. Moreover, minus RT controls were prepared for each sample using the identical procedure except for the omission of the enzyme.

### Real-time PCR

The real-time PCR was performed using the MX3005P device (Stratagene, La Jolla, CA, USA). The reactions contained 1× Brilliant SYBR Green QPCR Master Mix (Stratagene), 30 nM ROX reference dye, each primer at 200 nM and prediluted cDNA (according to 10 ng total RNA) in a 25 μl reaction. After an initial denaturation step at 95°C for 10 min amplification was performed with 40 cycles of denaturation at 95°C for 30 s, annealing at 60°C for 40 s and extension at 72°C for 40 s. Amplification was followed by a melting curve analysis to confirm PCR product specificity.

No signals were detected in no-template controls. Minus RT controls were run for those primer pairs, that have been shown to give a PCR product of same size and melting temperature in initial test experiments with genomic DNA (0.1–50 ng/well). No signals were detected in the minus RT controls.

The experimental threshold (Ct) was calculated using the algorithm enhancements provided by the MxPro Mx3005P v3.00 software: amplification based threshold, adaptive baseline, moving average. All samples were run in duplicate and the mean value of each duplicate was used for all further calculations.

During optimization of the protocol PCR products were loaded on 2 % agarose gels to confirm specificity of amplification and the absence of primer dimer formation. PCR efficiencies were estimated by running standard curves with the above described pool cDNA (5 points, cDNA amount between 25 ng and 0.04 ng according to initial total RNA concentration).

### Determination of reference gene expression stability

Two publicly available software tools, geNorm [19] and NormFinder [11] were used to analyze gene expression stability. Both tools require the transformation of Ct values to linear scale expression quantities. The average Ct values of the duplicates were therefore exported into Microsoft Excel from the Mx3005P software. Ct values were converted into relative quantities (Q) via the delta-Ct method by the formula Q = (E)^dCt^, with dCt = Ct of the highest abundant sample-Ct of the sample and E (efficiency) as determined by linear regression (calculated by the Mx3005P software). The quantities were then imported into geNorm software [50] (version 3.4), which was used as described in its manual. Twenty data points were used per time point (five biological replicates per treatment). Each of the three time points was analyzed separately. GeNorm calculates a gene-stability measure M, which is the average pairwise variation of a particular gene with all other control genes. Genes are ranked according to the determined M value from the least stable (highest M value) to the most stable (lowest M value).

Moreover, the calculated quantities were entered into a second software tool, NormFinder [51]. NormFinder estimates the overall expression variation of the candidate normalization genes and the variation between sample subgroups of the sample set using a model-based approach [11]. According to the resulting stability value the candidate reference genes can be ranked based on their expression stability.

A workflow diagram schematically depicting the process of stable gene selection is provided (Additional file 1).

### Calculation of the expression ratio of candidate reference genes in the different treatment groups versus control

The relative expression software tool (REST^©^) [52,53] was adopted to calculate expression ratios. This software allows for a group-wise comparison of expression differences. Moreover, the expression ratio results are tested for significance by a Pair Wise Fixed Reallocation Randomisation Test ^©^. Out of the several software versions, REST-MCS was chosen, because this allows the comparison of up to six experimental conditions against one reference condition for up to ten samples per group. Each of the three time points was analyzed separately. The experimental group (iii), i.e. sham operated, served as the reference condition. The two most stable genes, as calculated in advance by geNorm, were set as reference genes. The absolute gene regulation values along with the corresponding standard error, calculated by the REST-MCS software, were then forwarded to Microsoft Excel to create the graph presented in Fig. 3. For comparison, the reference condition, i.e. sham operated, was assigned a value of 1.

### Calculation of the relative expression of *B2m *and *Mac-1 *in the different treatment groups

Ct values were converted into relative quantities (Q) via the delta-Ct method by the formula Q = (E)^dCt^, with dCt = Ct of the highest abundant sample-Ct of the sample and E (efficiency). Relative quantities were normalized by dividing by the normalization factor calculated by geNorm from the two most stable genes. Normalized relative quantities were then rescaled by dividing by the arithmetic mean of the normalized relative quantities of the control group (sham w/o). The calculated values were imported into Prism 4 program (GraphPad Software, Inc., San Diego, CA) to generate the scatterplots presented in Fig. 4 and Fig. 6.

## Authors' contributions

KL designed and performed the qPCR experiments, was responsible for data analyses and writing the manuscript. RJ and HS carried out all animal surgery, minocycline treatment, including tissue sampling. They participated in RNA purification. UE primarily established the ACA experimental model and supervised RJ and HS in surgery. GK conceived the study, performed the immunohistochemistry and critically reviewed the manuscript. All authors read and approved the final manuscript.

## Supplementary Material

Additional file 1 **Workflow schema for reference gene selection**. The diagram illustrates the process of stable gene selection for normalization purposes in relative quantification. Workflow is given for two software programs, geNorm and NormFinder. The scheme is based on [11,19] as well as the accompanying software manuals [55,56]. For the principle of these programs see [11,19]. Transformation of raw Ct values into quantities can alternatively be performed using standard curves. The use of five to ten candidate reference genes is strongly recommended [56]. (HKG: Abbr. housekeeping gene). Click here for file
