# Supplementary material for: Selection of reference genes for quantitative real-time PCR in a rat asphyxial cardiac arrest model
Source: BMC Mol Biol. 2008 May 28;9:53. doi: 10.1186/1471-2199-9-53 (PMC2430208; doi:10.1186/1471-2199-9-53)
Supplement: Additional file 1 — Workflow schema for reference gene selection. The diagram illustrates the process of stable gene selection for normalization purposes in relative quantification. Workflow is given for two software programs, geNorm and NormFinder. The scheme is based on [11,19] as well as the accompanying software manuals [55,56]. For the principle of these programs see [11,19]. Transformation of raw Ct values into quantities can alternatively be performed using standard curves. The use of five to ten candidate reference genes is strongly recommended [56]. (HKG: Abbr. housekeeping gene). [file 1471-2199-9-53-S1.pdf]

1. *Choose ≥ 8 potential reference genes*

- Should belong to different functional classes, to reduce the chance of co-regulation
- Should best cover a range of expression levels
- Carefully validate all primers/assays (e. g. specificity, efficiency)

2. *Perform thermal cycling*

- Collect Ct values for each sample and reference gene using the instrument's software
- Measure duplicate or even triplicate values per sample

3. *Calculations*

- Calculate for each sample and reference gene the arithmetic mean of Ct values
- Calculate for each sample and reference gene relative quantities (delta-Ct method):
  $Q = (E)^{dCt}$
  dCt = Ct of highest abundant sample – Ct sample
  E = efficiency, Q = quantity

4. *Run software geNorm* *and/or* *Run software NormFinder*

geNorm

- Arrange data in an expression data matrix (Excel file)

	HKG 1	*HKG 2*	*HKG 3*	*HKG 4*	*HKG 5*	*HKG 6*	*HKG 7*	*HKG 8*
**sample 1**								
**sample 2**								
**.**								
**.**								
**.**								
**sample n**								

- Run *Manual analysis* or *Automated analysis*

Manual analysis:

M-values of the **least** and **most** stable gene are highlighted
↓
Eliminate worst scoring HKG (highest M-value)
↓
Run calculation again
⇘ *end up with the two best references genes*

Automated analysis:

Two charts are generated:
- Average expression stability chart:
  - Genes are ranked according to increasing stability (two most stable genes at the right)
- Pairwise variation chart:
  - Indicates pairwise variation between two sequential normalization factors
  - Helps to choose the number of references genes necessary for reliable normalization

NormFinder

- Arrange data in an expression data matrix (Excel file)

	sample 1	*sample 2*	*sample3*	*...*	*sample n*
**HKG 1**					
**HKG 2**					
**.**					
**HKG n**					
The last row may contain a group identifier (integer).					

- Run programm

- Each candidate reference gen is assigned a "stability value" (best gene – lowest value)
- Best gene highlighted
- If group identifiers were included, best combination of two genes is given additionally
